# Are there distinct dimensions of apathy? The argument for reappraisal

**DOI:** 10.1016/j.cortex.2022.01.001

**Published:** 2022-04

**Authors:** Shannon S. Dickson, Masud Husain

**Affiliations:** a Department of Experimental Psychology, University of Oxford, Oxford, UK; b Nuffield Department of Clinical Neurosciences, John Radcliffe Hospital, Oxford, UK

**Keywords:** Apathy, Dimensions, Assessment, Framework, Definition

## Abstract

Apathy is widely accepted to be a multidimensional syndrome. Assessment scales typically probe one or more dimensions but there is no consensus on the precise nature of these domains. Existing major theoretical frameworks include cognitive, behavioural, and emotional dimensions of apathy. While a social domain has also been suggested, it is far less well studied. Here we argue that although most assessment scales have been developed with these theoretical frameworks in mind, few findings actually support the existence of some of the dimensions that have been proposed, with the evidence for separation of cognitive and behavioural dimensions particularly lacking. In our opinion, although there is evidence for behavioural and emotional domains of apathy, the contention that there might be a separate dimension of cognitive or executive apathy is far less robust. Further, while there is some evidence for a social dimension of apathy, this has not been investigated sufficiently to make any definitive conclusion. We argue that there is a pressing need to reconsider different domains of apathy using robust analyses of proposed theoretical dimensions.

## 1 Introduction

The clinical syndrome of apathy is a common condition, now recognised to occur across a wide range of brain disorders (Le Heron, Holroyd, Salamone, & Husain, 2019; Marin, Wilkosz, 2005; Starkstein & Leentjens, 2008). Prevalence rates vary depending upon the instrument used for assessment but some recent estimates suggest apathy might occur in as many as 49% of patients with Alzheimer's Disease (AD) (Zhao et al., 2016), 40% with Parkinson's Disease (PD) (den Brok et al., 2015), and 38% of individuals with late-life depression (Yuen et al., 2015). The syndrome is associated with greater cognitive decline, increasing disease severity, poorer quality of life and more caregiver burden in AD and PD (Chen et al., 2018; Martinez-martin et al., 2015). It is therefore emerging as an important factor to stratify severity and prognosis in many different patient groups. Moreover, because there is currently no established licensed treatment for the syndrome, there is considerable interest in developing new therapies and robust measures to index change in apathy. Although there is overwhelming evidence for the existence of an apathy syndrome, in our opinion what is far more questionable is the evidence for the different dimensions of apathy that have been proposed.

### 1.1 Apathy frameworks and assessment scales

Most researchers appear to agree that apathy is a *multidimensional* syndrome, but the precise details of these dimensions vary according to different theoretical positions. One highly influential conceptual framework has operationalised apathy as a *“loss of motivation unattributable to emotional distress, cognitive impairment, or diminished consciousness”* (Marin, 1990). In this formulation, apathy is considered to exist in three potentially dissociable components of goal-directed behaviour: diminished productivity (*behaviour*), diminished goals (*cognition*), and diminished emotional responses to success or failure (*emotion*). The distinction of apathy from similar conditions, such as depression or anhedonia, is also made clear.

A second influential theoretical framework also proposes three domains of apathy but in this perspective the dimensions are cognitive, emotional-affective, and auto-activation, with the latter highlighting the importance of self-initiated activity (Levy & Dubois, 2006). The key difference from Marin's theoretical perspective in this account is a move away from separate cognitive and behavioural dimensions and the introduction instead of *“auto-activation”* deficits that subsumes both of them. Another notable difference is the replacement of cognitive apathy with deficits in *executive function* that Marin had excluded as a cause of apathy. Some investigators have also proposed the existence of a social domain (Ang, Lockwood, Apps, Muhammed, & Husain, 2017; Sockeel et al., 2006; Stuss, van Reekum, & Murphy, 2000) which has been incorporated into recent clinical criteria (Robert et al., 2018). However, researchers disagree on which of these perspectives regarding the dimensions of apathy is correct.

**Fig. 1:**
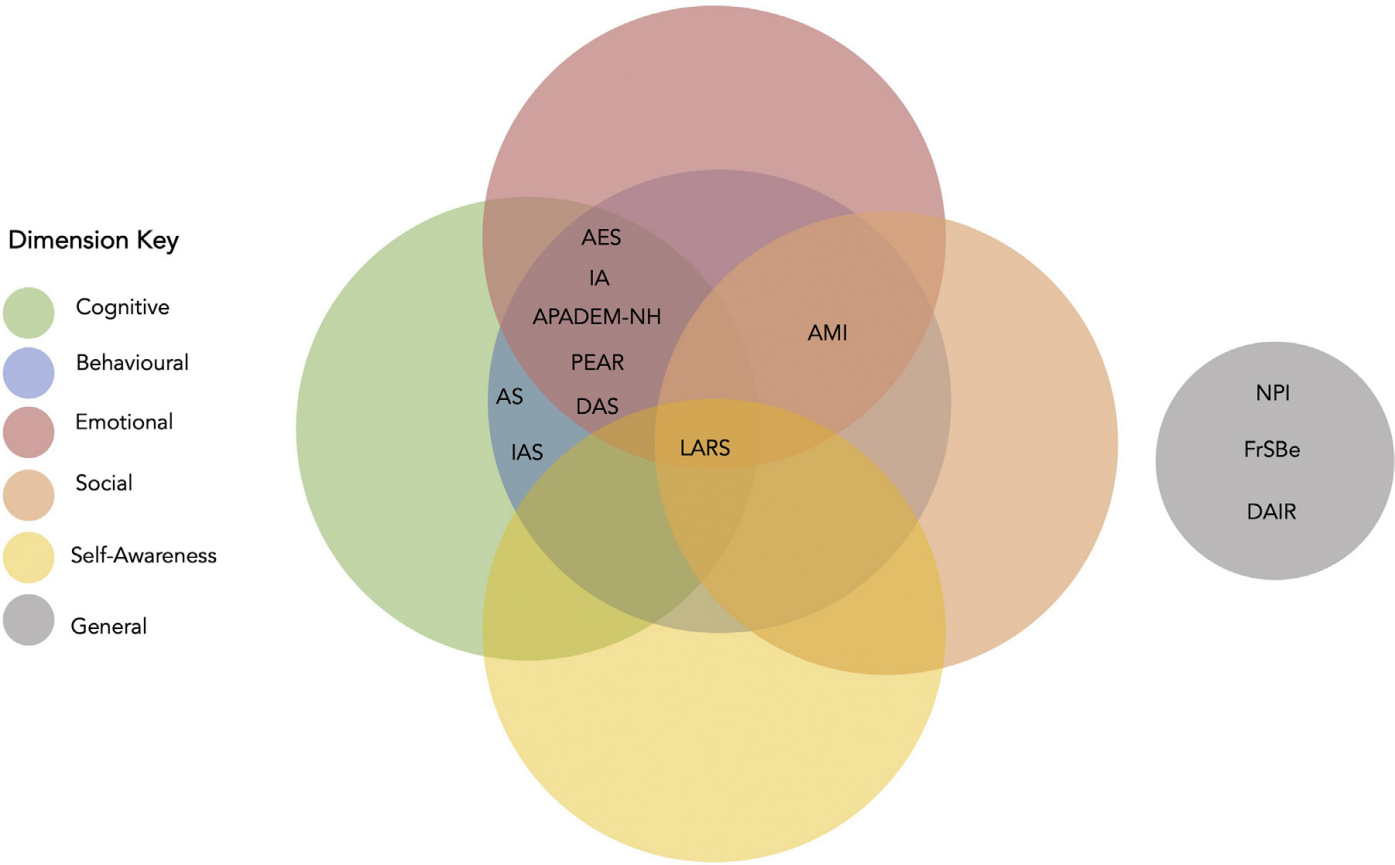
Venn-Diagram of the major apathy dimensions assessed in different apathy scales. Note: AS: apathy scale; IAS: irritability apathy scale; AES: apathy evaluation scale; IA: apathy inventory; APADEM-NH: apathy in institutionalised persons with dementia–nursing home; PEAR: Pearson environment apathy rating; LARS: Lille apathy rating scale; DAS: dimensional apathy scale; AMI: apathy motivation index; FrSBe: frontal system behaviour scale; DAS: dementia apathy rating scale; NPI: neuropsychiatric inventory.

In parallel with the development of these conceptual frameworks, several different apathy scales have been constructed (Table 1), often reflecting the theoretical dimensions of the syndrome that investigators subscribe to (Fig. 1). Marin's Apathy Evaluation Scale (AES) is the earliest and perhaps the most influential formalised method (Marin, Biedrzycki, & Firinciogullari, 1991). The AES measured apathy along cognitive, behavioural, and emotional dimensions and has inspired many scales with similar structures (Robert et al., 2002; Sockeel et al., 2006; Starkstein et al., 1992). For example, Starkstein et al. (1992) Apathy Scale (AS) is a 14-item abridged version of the AES having six items in common with it, primarily measuring cognitive and behavioural apathy. The Apathy Inventory (IA) is a far briefer instrument but it consists of one item each for cognitive, behavioural, and emotional apathy (Robert et al., 2002). By contrast, the Lille Apathy Rating Scale (LARS), which also owes some of its heritage to the AES, is far more extensive, comprising 33-items across nine domains which are lack of interest, extinction of novelty seeking, reduced motivation, lack of concern, poor social life, reduction in everyday productivity, lack of initiative and blunted emotional response (Sockeel et al., 2006).

**Table 1:** Summary of apathy scales and the domains assessed in chronological order.

Apathy scales	Administration	N-items	Domains assessed
*Multidimensional*			
Apathy Evaluation Scale (AES-S/I/C) Marin et al. (1991)	AES-S: self-report AES-I: informant based AES-C: clinician administered	18-items	
Apathy Scale (AS) Starkstein et al. (1992)	Self-report, informant, and clinician administered	14-items	
Apathy Inventory (IA) Robert et al. (2002)	Self-report and informant versions	3-items	
Irritability Apathy Scale (IAS) Burns, Folstein, Brandt, and Folstein (1990)	Informant interview	5-items	
Apathy in Institutionalised Persons with Dementia (APADEM-NH) Lanctôt et al. (2017)	Informant interview	26-items	
Lille Apathy Rating Scale (LARS) Sockeel et al. (2006)	Self-report/patient interview, Caregiver based	33-items	
Dimensional Apathy Scale (DAS) Radakovic and Abrahams (2014)	Self-report	24-items	
Apathy Motivation Index (AMI) Ang et al. (2017)	Self-report	18-items	
Pearson Environment Apathy Rating (Jao et al., 2016)	Clinician observation	12-items	
*Unidimensional*			
Neuropsychiatric Inventory (NPI) Cummings et al. (1994)	Patient/informant interview	16-items	
Frontal System Behaviour Scale (FrSBe) Grace et al. (1999)	Self-report, informant-based, clinician interview	46-items, 14 for apathy	
Dementia Apathy Rating Scale (DAIR) Strauss and Sperry (2002)	Informant interview	1-item for apathy answerable by “yes” or “no”. Follow-up questions on frequency and severity of symptom asked if response is “yes”.	

Cognitive.

Behavioural.

Cognitive-Behavioural.

Emotional.

Social.

Self-Awareness.

General.

Levy & Dubois' (2006) conceptualisation of apathy is a theoretical view based on cognitive neuroscience and focal lesion perspectives. The first empirical test of this framework was with the development of the Dimensional Apathy Scale (Radakovic & Abrahams, 2014). The DAS has 24-items covering executive, (behavioural/cognitive) initiation, and emotional axes of apathy. Radakovic and Abrahams later formulated their own Dimensional Apathy Framework (DAF), specifying initiation apathy (cognitive and behavioural), executive apathy, and emotional apathy (Radakovic & Abrahams, 2018). The DAF is quite closely aligned to Levy and Dubois' theory with the exception of an additional self-awareness component that operates across all of the dimensions.

Several other independent apathy scales have also been developed, for example the Apathy Motivation Index (AMI), with 18-items distributed evenly across three domains (Ang et al., 2017). In this framework, Behavioural activation (BA) is an individual's self-initiated purposeful behaviour, emotional sensitivity (ES) relates to an individual's positive and negative affect, and social motivation (SM) captures engagement in social interactions. Some scales have been developed for use in specific populations, such as the Apathy in Institutionalised Persons with Dementia (APADEM-NH) (Lanctôt et al., 2017), which follows a triadic structure including deficits of thinking and self-generated behaviour, cognitive inertia and emotional blunting. The Pearson Environment Apathy Rating (PEAR) (Jao, Algase, Specht, & Williams, 2016) consists of an Environment subscale, examining the relationship between apathy and the environment, and an Apathy subscale, assessing facial expression, eye contact, physical engagement, purposeful activity, verbal tone and verbal expression. In addition, an assessment of apathy is also made, albeit in only one general syndromic dimension, in broader clinical tools such as the Neuropsychiatric Inventory (NPI) (Cummings et al., 1994), the Frontal Systems Behaviour Scale (FrSBe) (Grace, Stout, & Malloy, 1999) and the Dementia Apathy Rating Scale (DAIR) (Strauss & Sperry, 2002).

### 1.2 A critique of the classic apathy frameworks

The original division of apathy into cognitive, behavioural, and emotional subtypes was based on observations of patients with neurological conditions (Marin et al., 1991). Similarly, Levy and Dubois' (2006) theory was formulated on insights from cognitive neuroscience and lesion data. Importantly, as we review below, these frameworks are not actually based on empirical evidence of clearly dissociable domains of apathy, but are rather theoretical formulations based on the authors' conceptualizations from the prior literature or observations. It is somewhat contentious then that most apathy instruments are based upon these two triadic models (Levy & Dubois, 2006; Marin et al., 1991; Mulin et al., 2011; Radakovic & Abrahams, 2014; Robert et al., 2002; Sockeel et al., 2006; Starkstein et al., 1992; Strauss and Sperry, 2002). While these syndromic conceptualisations of apathy have considerably advanced our understanding of the condition, closer examination of the factorial structure of many apathy scales actually reveals different dimensions to the ones that were originally proposed.

Here we critically examine the literature on theoretical frameworks for different domains of apathy. Most of the empirical data that have been used to support claims for separate dimensions come from studies which report results from the use of apathy scales. We assess whether these actually reveal the dimensions they are supposed to dissociate when the data have been scrutinised by rigorous factor/structural analysis. We conclude that although there is evidence for behavioural and emotional blunting domains of apathy, the contention that there might be a separate domain of cognitive or executive apathy is less robust on the basis of the extant literature. Further, while there is some evidence for a social dimension of apathy, this domain has not been investigated sufficiently in previous work to make any definitive conclusion. For the interested reader, detailed reviews of psychometric properties of apathy scales are found elsewhere (Clarke et al., 2011; Radakovic, Harley, Abrahams, & Starr, 2015).

## 2 What is the evidence for separate apathy dimensions?

### 2.1 Revisiting Marin's cognitive, behavioural, emotional framework

The contention that apathy might have separate, dissociable components originates from Marin's pioneering work in this field. Marin developed the AES out of a need for a formalised means of assessing apathy in clinical populations. To this end, his primary goals were to distinguish apathetic people from non-apathetic people, to separate apathy from depression and lastly to discriminate between different disorders based only on the degree to which they are apathetic (Marin et al., 1991) But the evidence that led him to the conclusion that there might be cognitive, behavioural and emotional domains of apathy is arguably insufficient.

The AES was developed to obtain perspectives from several sources: the clinician (AES-C), informant (AES-I), and the patient themself (AES-S). It was originally validated in a mixed sample of 123 patients with AD, hemispheric stroke, depression, and healthy individuals. Marin devised eight, five, two and three questions for cognitive, behavioural, emotional, and general apathy respectively. However, a principal component factor analysis (PCA) actually revealed a predominantly single-factor structure for the AES with item loadings depending on how it was administered (Marin et al., 1991). The main factor across the different administrations accounted for between 32 and 53% of the variance and represented a general apathy. Items for the two AES-C ratings and the AES-S rating were very similar, except for one emotional item (*“when something good happens, s/he gets excited”*). Similarly, all but two items loaded onto this general factor on the AES-I rating. Thus, all versions of the AES consistently produced a main general apathy factor. The second factor related to “interest” or “curiosity” but accounted for just 5–10% of the variance across administrations. Only one of the AES-C reports found a third factor accounting for 7–8% of the variance that represented lack of insight/concern and needing external structure for daily routines (Marin et al., 1991). Despite slight variations in item loadings, these general and interest factors are considerably different to the discrete cognitive, behavioural, or emotional dimensions advocated by Marin.

In a similar manner, in a mixed dementia sample Clarke et al. (2007) reported two factors for the AES-C and AES-I that represented “general apathy” and “interest” accounting for 51.1% and 54.4% of the total variance respectively. They also provided evidence for a “general apathy” and “other” factor for the AES-S accounting for a collective variance of 43.3%. Examples of items belonging to the “other” factor are *“Do you get things done during the day?”*, *“Do you put little effort into anything?”* and *“Are you less concerned about your problems than you should be?”.* It is not clear what makes these three items different to the general factor, but the loadings were moderate to low at .546, .436, and .230 (Clarke et al., 2007).

This pattern of a main general factor and smaller “interest” factors has also been found in patients with stroke, subarachnoid haemorrhage, and psychosis (Sagen et al., 2010), starkly contrasting with the factors Marin originally proposed. Hsieh, Chu, Cheng, Shen, and Lin (2012) did find a three-factor structure but none pertained to the original dimensions proposed by Marin. The largest factor they found accounted for 29% of the total 49% of explained variance and included items from *both* interest (cognitive) and initiative (behaviour) domains. The remaining items actually related to drive and self-awareness (11%), and social aspects of apathy (9%), but not emotional apathy.

Despite its theoretical divergence from what has been found in practice, including in his own original study, Marin's framework remains largely unchallenged. The continued use of the AES and some of its derivatives is perhaps because they benefit from good psychometric properties when measuring apathy as a whole construct (Clarke et al., 2011; Marin et al., 1991) undoubtedly making them a valuable tool for clinical or diagnostic purposes when a rapid assessment is necessary. However, as we have seen, relatively few studies have considered if the AES is adequately assessing the apathy spectrum. Of those reports that do address this question, the classic triadic structure is rarely supported by the evidence presented. As we go on to discuss, subsequent scales that owe their heritage to Marin's AES also reveal a dimensionality that contradicts the original framework.

The AS, which was designed by Starkstein and colleagues to assess apathy in PD, is one example of this. Surprisingly, no factor analysis was performed in their validation study (Starkstein et al., 1992), but subsequent investigations that do provide this have reported both three-factor (Kay, Kirsch-Darrow, Zahodne, Okun, & Bower, 2012) and two-factor models (Lopez et al., 2019; Pedersen et al., 2012). While one study of the AS lends support to a triadic model of cognitive, behavioural and emotional apathy (Kay et al., 2012), a later analysis found this account suffered from factor overextraction indicated by correlations between the cognitive and behavioural latent factors (Lopez et al., 2019).

PCA of the AS in PD has shown that cognitive-behavioural and general apathy factors account for 24.2% and 15% of the total variance in turn (Pedersen et al., 2012). One item, which asked if patients are concerned about their condition, was removed due to poor item–total correlations. The cognitive-behavioural factor included items relating to goals, plans, and interest as well as effort and energy, whereas the general apathy factor appeared predominantly emotional in type with some aspects of general motivation. The two factors were uncorrelated, suggesting in this case that there is evidence for cognitive, behavioural, and emotional *types* of apathy, but little evidence that the former two are dissociable dimensions as originally envisaged. A major limitation of this study is that factor analysis is more appropriate than PCA when investigating latent structure of a broader construct (Conway & Huffcutt, 2003; Schmitt, 2011). Furthermore, the model did not satisfy multiple indices of fit. However, exploratory factor analysis of the AS has recently revealed largely identical factor compositions to those previously found by Pedersen et al. (2012) in PD (Lopez et al., 2019).

Lopez et al. (2019) labelled these two factors *Motivation-Interest-Energy* and *Indifference,* each accounting for 40.9% and 13.2% of the total variance. Motivation-Interest-Energy was in near-total agreement with Pedersen et al.'s (2012) cognitive-behavioural factor, as was Indifference with their general apathy factor, representing blunted emotion and lack of concern. The only difference in the two models is the removal of item 2 (*“Are you concerned about your condition?”*), item 13 (*“Are you neither happy nor sad, just in between?”*) and 14 (*“Would you consider yourself apathetic?”*) due to their high cross-loadings. This new AS factor structure was not verified against formal diagnostic criteria or other independent measures, but self-reported apathy, which is vulnerable to bias. Nevertheless, with the modifications (removal of items 3, 13, and 14) an acceptable model fit was achieved across three different indices for factors representing an amalgamation of cognitive/behavioural apathy and separate emotional apathy in PD (Lopez et al., 2019), mounting further evidence against Marin's triadic framework.

The LARS is one instrument that has proved sensitive to the multidimensional nature of apathy. In the original validation, it was possible to dissociate cognitive, behavioural, and emotional apathy in PD patients (Sockeel et al., 2006). The authors found four factors they termed *intellectual curiosity* (corresponding to cognitive), *action initiation* (corresponding to behavioural), *emotion* and *self-awareness* (Sockeel et al., 2006). Intellectual curiosity (IC) comprises interest, novelty seeking, motivation, and also social life; action initiation (AI) includes everyday productivity and initiation; while emotion relates to emotional blunting and lack of concern. This description of cognitive, behavioural, and emotional elements is strikingly similar to Marin's proposed goal-directed cognition, goal-directed behaviour, and emotional apathy and suggests they are indeed dissociable dimensions, at least on the basis of the responses to the LARS (but see below for further analysis). The importance of a self-awareness dimension in apathy has also been highlighted by the lack of correspondence in patient and caregiver reports on the IA (Robert et al., 2002). Previously, it was incorporated into cognitive or general apathy factors as a single question (Marin et al., 1991) but now exists as an independent dimension in the LARS.

These four factors in the LARS accounted for a good portion of the variance (65%). Furthermore, the global scores for the LARS and AES correlated strongly (r = .87), driven by higher correlations between the AES and IC (r = .84) and AI domains of the LARS (r = .65) and lower correlations with the emotional (r = .44) and self-awareness domains (r = .15) (Sockeel et al., 2006). These independent factors for IC and AI, coupled with their significant associations with the AES, might at first sight appear to support two unique dimensions of cognitive and behavioural apathy. However, these two factors also correlated with one another significantly (r = .656). Moreover, emotional blunting was closely correlated to IC (r = .435). Given these close correlations between the three main factors it would be difficult, we argue, to confidently advocate them as truly separate domains.

In fact, the authors themselves argue for a unidimensional structure of the LARS due to IC accounting for most of the variance (34%), although they do not report variances for the remaining factors (Sockeel et al., 2006). A later study from Zahodne et al. (2009) offers some support for this one dimensional proposal, reporting significant associations among all four factors. As in the original study, IC and AI were most strongly correlated (r = .506), followed by IC and Emotion (r = .356) and AI and Emotion (r = .268). Self-awareness was also significantly correlated with IC (r = .322) and AI (r = .251) but not with Emotion, whereas it was entirely independent before (Sockeel et al., 2006).

A Spanish version of the LARS that was validated in PD patients describes the same original factors as Sockeel et al. (2006) although represented by different constituent items (García-Ramos, Villanueva Iza, Catalán, Reig-Ferrer, & Matías-Guíu, 2014). They found everyday productivity, interest, and initiative loading on IC; novelty seeking, motivation, and social life loading on Emotion; emotional responsivity and self-awareness comprised SA; and only concern contributing to AI. In fact, there is very little overlap in the details of these factors compared to what was found before. In particular, IC includes more behaviourally oriented questions from the initiation and everyday productivity subscales than cognitively oriented questions. Moreover, there are no overtly behavioural questions included in the AI factor which it is supposed to represent. Meanwhile, the Emotional factor appears more in line with Sockeel et al.'s (2006) IC factor but could arguably represent the social elements of apathy too.

In schizophrenia patients, the LARS has a yet another factor structure (Yazbek et al., 2014). Not all subscales were considered in the resultant factors: *Novelty and Social Life* was the main factor (23%); followed by initiative and voluntary action making *Behavioural Involvement* (16.7%); emotional responsivity comprised *Emotional Involvement* (12.7%); and *Judgement Skills* included interest and self-awareness (10.1%). Interestingly, everyday productivity loaded on the first and third factors (but not Behavioural Involvement, which is like AI) and concern loaded on all factors leading to both subscales being excluded from the factors. The notion of productivity and concern were previously quite central to Marin's (1991) behavioural and cognitive domains but did not offer any discriminatory value in this case. Although the composition of these factors is different to previous findings (García-Ramos et al., 2014; Sockeel et al., 2006) cognitive (Judgement Skills), behavioural (Behavioural Involvement), and emotional dimensions (Emotional Involvement) are present. The authors argue that Novelty and Social Life also characterise a cognitive dimension but considering that this component comprises just novelty seeking and social life, a social dimension is equally plausible. Among the factors there was only a weak correlation between Novelty and Social Life. In schizophrenia, it seems that cognitive, behavioural and emotional dimensions of apathy are dissociable in the form of Judgement Skills, Behavioural Involvement, and Emotional Involvement whereas they are not consistently dissociable in other groups.

The nature of cognitive and behavioural questions on the LARS might partially account for the close association of Sockeel et al.'s (2006) IC and AI factors. As previously mentioned, in regard to the AES, the questions that have been designed to assess apathy are not specific enough to account for what is meaningfully different between cognitive and behavioural aspects of apathy. This is true for the LARS too. For example: *“What do you do to keep yourself busy?”* and *“When you decide to do something, are you able to make the effort easily?”* are cognitive items comparable to *“What do you get up to during the day?”* and *“Do you decide to do things, or does someone have to push you a little?”* that are behavioural items. The correlation between IC and Emotion can be similarly explained, illustrated by the resemblance between *“When you don't manage to do something, do you try to find other solutions”* and *“When something's not working or when something unexpected happens, do you think about finding a solution?”* that belong to the cognitive and emotional subscales, respectively. The result is that patients or family members who respond to these questions may not immediately understand how cognitive and behavioural apathy symptoms should be considered differently.

Even if cognitive and behavioural apathy are theoretically distinct, this separation may not exist in practice since assessments rely on what is observable, either by informants or by direct observation of the clinician. It is easy to appreciate how cognitive interest and behavioural initiative are highly related. Presumably, if a person is disinterested in certain hobbies, they are less likely to initiate the relevant actions. In the same way, if they lack the initiative to partake in their hobbies, then over time they will become indifferent to them and their interest wanes. Many of the items from the AES and other scales designed to assess cognitive and behavioural apathy separately are not nuanced enough to capture this distinction.

While behavioural items ask the extent to which people actually engage in their interests, cognitive items assess the importance they place on these interests and goals. This difference in emphasis is not easily distinguishable to a layperson, such as the patient or their caregiver, or even a clinician with limited time for assessment. For instance, in the AES, the cognitive item *“S/he is interested in things”* is not dissimilar to the behavioural counterpart *“S/he spends time doing things that interest her/him”.* Likewise, the difference between the cognitive item *“Getting things done during the day is important to her/him”* and the behavioural item *“S/he gets things done during the day”* is extremely subtle. A patient might be capable of saying they would like to read but lack the initiative to do this. However, a clinician cannot always rely on a patient having adequate insight of their internal states, as demonstrated by the low reliability of the patient based reports (Robert et al., 2002). In such circumstances, how can a caregiver or clinician decipher if reading is important to the patient if they never read?

Further, being “interested in things” is a rather vague question that a cognitively impaired, apathetic person might not be capable of interpreting. Given the nature of the questions, the responses of a patient – or indeed their caregiver – could easily conflate cognitive and behavioural apathy as measured by the AES and its derivatives. Arguably, this distinction between cognitive and behavioural apathy is extremely difficult to capture through questionnaires, although there might be a role for using experimental techniques to distinguish between the putative cognitive and behavioural dimensions of apathy.

### 2.2 Levy and Dubois' cognitive, auto-activation, and emotional-affective framework

Do alternative theories of apathy stand up to scrutiny? The lack of empirical testing of Levy and Dubois's (2006) framework makes it difficult to answer this question conclusively. The DAS (Radakovic & Abrahams, 2014) is the only apathy scale developed following their guideline of executive, auto-activation, and emotional-affective subtypes. In healthy people, four preliminary factors have been demonstrated which the authors termed *Executive, Emotional*, *Cognitive Initiation*, and *Behavioural Initiation* (Radakovic & Abrahams, 2014). Executive apathy, pertaining to difficulties in organisation, attention, and planning was the largest factor (12.9%); followed by Emotional (6.2%); while the initially separate Cognitive Initiation (5.3%) and Behavioural Initiation (4.6%) were ultimately combined for thematic reasons. Although these factors approximate to Levy and Dubois' apathy dimensions, they actually explained little of the variance (28.9%) on the DAS overall in this healthy group. Correlations between the Executive and the Cognitive/Behavioural Initiation subscales (r = .648), and the Cognitive/Behavioural Initiation and Emotional subscales (r = .236), also imply that the behavioural dimension shares some qualities of cognitive and emotional apathy and are interdependent to some degree.

Slightly stronger evidence for executive, emotional, and initiation domains comes from a study validating an Italian version of the DAS in healthy people (Santangelo et al., 2017). PCA revealed three domains, the first and largest relating to executive organisation, attention, and planning abilities (23.94%); the second relating to initiation of thoughts and behaviours (8.95%); and the third and smallest once more relating to the processing and expression of emotion (7.09%). Still, there were cross-loadings with empathy items on both the executive and emotional factors. Overall, the Italian DAS showed a high degree of convergence with the original factors (Radakovic & Abrahams, 2014), but with greater reliability (Santangelo et al., 2017). Self-report, informant-based, and brief versions of the DAS and its subscales positively correlate with the AES, suggesting good concurrent validity (although consider the limitations of the AES discussed) (Radakovic, Starr, & Abrahams, 2017, 2018, 2020). Further, the DAS is more sensitive to apathetic subtypes than the AES, identifying significantly more AD and PD patients with Executive and/or Initiation apathy (Radakovic et al., 2017, 2018).

On the other hand, the emotional subscale of the DAS has proved less reliable as a singular dimension (Radakovic et al., 2017, 2018). It showed no correlation with the AES total or emotional subscale in one study (Santangelo et al., 2017), but was at least independent from measures of depression (Radakovic et al., 2017, 2018; Santangelo et al., 2017). Across some investigations emotional apathy was more commonly found in patients with AD compared to others with PD and ALS (Radakovic & Abrahams, 2018; Radakovic et al., 2017, 2018). So, this heterogeneity across neurodegenerative diseases might partly explain the unreliability of an Emotion dimension as measured by the DAS (Radakovic et al., 2018).

In the case of the DAS, dissociating cognitive and behavioural dimensions was possible only by changing the core definition of cognitive apathy to denote executive functions and abilities. Items on the Executive subscale, such as *“When doing a demanding task, I have difficulty working out what to do”* were subsequently considered to characterise cognitive apathy. This is very similar to Levy and Dubois' “cognitive processing” apathy dimension but distinctively different to Marin's notion of reduced goal-directed behaviour that is primarily characterised by being “interested” in things (Levy & Dubois, 2006; Marin et al., 1991). Some studies have demonstrated an association between poor executive functioning and apathy in healthy elderly people (Montoya-Murillo, Ibarretxe-Bilbao, Peña, & Ojeda, 2019) neurological disorders like PD (Zgaljardic et al., 2007) and AD (McPherson, Fairbanks, Tiken, Cummings, & Back-Madruga, 2002), supporting the possibility that executive dysfunction might be one mechanism contributing to apathy in these groups.

Theoretically, however, there are limitations to the inclusion of an executive dysfunction as a *dimension* of apathy. While it might be the case that the abilities necessary for generating purposeful action such as planning, organisation, and goal-maintenance, are typically the same processes affected by cognitive abnormalities, apathy is not fully accounted for by these deficits (Brodaty, Altendorf, Withall, & Sachdev, 2010; Marin et al., 1991; McPherson et al., 2002). In other words, not all patients with those cognitive deficits would be classed as apathetic (Marin, Butters, Mulsant, Pollock, & Reynolds, 2003; Robert et al., 2006). Therefore, it is potentially problematic that the core component of the DAS is Executive (function), presenting the possibility of falsely classifying someone as apathetic on the basis of this dimension alone.

### 2.3 Other apathy scales

Is there evidence of separate cognitive, behavioural, and emotional apathy domains from any other apathy scales? Unfortunately, most of the remaining scales that have been developed are not optimally constructed to assess syndrome dimensionality. One such example is the IA (Robert et al., 2002) which has been validated in a mixed sample of healthy people and neurocognitive disorders. Each subscale (interest, initiation, emotional) is assessed by just one question because this is designed to be a brief test, but this makes construct analysis unfeasible. Furthermore, only the interest and initiation subscales of the AI-caregiver version correlated with the NPI-Apathy score. The lack of evidence for an emotional subscale is potentially explained by the different apathy profiles present across the neurocognitive disorders that were studied (Robert et al., 2002), and that are also observed with other scales like the LARS (García-Ramos et al., 2014; Sockeel et al., 2006; Yazbek et al., 2014) and the AMI (Ang et al., 2017). Regardless, the NPI should not be used as a benchmark since it is a unidimensional tool itself and tells us nothing about possible different apathy dimensions.

Another example is the Irritability Apathy Scale which has only five questions for apathy summarised as *Loss of interest*, *Lies Around, Not as Active*, *Keeps Busy*, and *Withdrawn* (Burns et al., 1990). Most of these questions are consistent with behavioural aspects of apathy, with two representing more cognitive features (Loss of Interest, Withdrawn). However, the authors did not provide evidence that these domains are dissociable. Both the DAIR (Strauss, Sperry, 2002) and the APADEM-NH (Agüera-Ortiz et al., 2015), developed specifically for dementia patients, were found only to represent a single apathy construct.

Thus, the evidence for the three dissociable domains – cognitive, behavioural and emotional – that were originally formulated by Marin is actually rather unsubstantiated in the literature, even in Marin's original work. Perhaps the most convincing evidence comes from the LARS, which did find different dimensions in several studies (García-Ramos et al., 2014; Sockeel et al., 2006; Yazbek et al., 2014). Although the composition of the factors identified varied depending on the sample, they broadly conform to cognitive, behavioural, and emotional apathy dimensions. Studies using the LARS also support an additional self-awareness factor, which was subsequently included in the Dimensional Apathy Framework introduced by the authors of the DAS (Radakovic & Abrahams, 2018). Even so, contrary to what Marin proposed, those studies show that cognitive and behavioural apathy dimensions are considerably interrelated.

### 2.4 The case for a new domain: social apathy

Are there alternative apathy domains to those previously studied? This question has been somewhat neglected in the current literature. Recent updates to the diagnostic criteria for apathy make the case for a “social interaction” domain (Robert et al., 2018), that leads to a loss of spontaneous or environmentally stimulated social interaction, disinterest in friends and family, or a preference for staying at home. Social apathy has not been widely sought as a separate dimension, probably due to the lack of a social component in the major apathy theoretical frameworks (Levy & Dubois, 2006; Marin et al., 1991; Radakovic & Abrahams, 2018). The earliest mention of a social dimension came from Stuss and colleagues who likened it to a lack of self-awareness of one's personal, social past, and present information (Stuss et al., 2000). Self-awareness did then emerge as a LARS factor but showed no correlation with the items assessing social life in particular. Rather, social life is factored into the cognitive domains of both the LARS and the AES (Marin et al., 1991; Sockeel et al., 2006).

Recent support for social apathy comes from the AMI, which revealed a *social motivation* domain (SM: engagement in social interactions) in addition to *behavioural activation* (BA: self-initiation of goal-directed behaviour) and *emotional sensitivity* (ES: emotional responsivity) in healthy people (Ang et al., 2017). Some significant correlations were present between SM and ES (r = .168) and between SM and BA (r = .330), although they are weak. When compared to the DAS, the SM dimension correlated moderately with all three subscales of the DAS (Executive: r = .14; Emotional: r = .22; Initiation: r = .53) (Ang et al., 2017). Given that the SM subscale includes some notion of initiation/activation (e.g., *“I start conversations without being prompted”*) and emotional investment in a situation (e.g., *“I enjoy doing things with people I have just met”*), it is perhaps not unreasonable to find such correlations.

In fact, it has recently been reported that the French translated-DAS emotion subscale can be partitioned into two further apathy subtypes in healthy people: an *individual emotional t*ype which relates to interpersonal emotional expression/stimulation; and a *social emotional* type relating to external interactions such as empathy and concern for others (M'Barek, Radakovic, Noquet, Laurent, & Allain, 2020). In addition, reducing the DAS emotional subscale to just the socially driven items led to a better fitting model of apathy than other variations to the emotional subscale (M'Barek et al., 2020). Despite the original authors not considering a distinct social dimension when developing the DAS, including this domain might potentially improve its performance if re-examined in other populations.

The AMI, which is adapted from the LARS, might also be a useful measure to detect social apathy. It has been shown to delineate different profiles of apathy domain across different groups. In a study from the original authors, social apathy (indexed by the SM factor) was more pronounced in PD patients compared to healthy older adults (Ang et al., 2017). Social apathy has also been observed in healthy people using the AMI in conjunction with an experimental effort-based reward task; those individuals with high scores on the SM subscale (indicating high social apathy) exerted less effort when the reward was given to another person compared to if it was given to them (Lockwood et al., 2017). This behavioural effect was uniquely related to social apathy.

Social apathy, as measured by the AMI, was also closely related to anhedonia, fatigue, and depression in a way that emotional apathy, for example, was not, again suggesting that it represents a dissociable domain (Ang et al., 2017, 2018). In a recent study of healthy individuals, SM was found to be the only apathy domain to correlate negatively with impulsivity, suggesting that an apathy–impulsivity axis is unique to the social domain, thereby further supporting a distinct status of this domain of apathy (Petitet et al., 2021). Importantly, apathy scales that exclude a social dimension, like the AES and its derivatives, will be unable to parcel out this nuanced association between apathy and impulsivity (Petitet et al., 2021). As yet though there are very few studies that have used this recently developed scale. Profiling social apathy and its associations in different patient groups will be an important focus for future investigation.

Overall, our analysis supports the inclusion of a social dimension in the new diagnostic criteria for apathy (Robert et al., 2018), consistent with a large body of work in cognitive neuroscience and neurology that has implicated specific brain regions and networks in human social interaction (Kennedy & Adolphs, 2012). At the very least, it is worthwhile to explore further the social domain of apathy considering the paucity of research to date that has addressed this dimension. The AMI might be a practical tool to investigate social apathy. It presents good psychometric properties and is capable of identifying apathy without the need for the contested cognitive dimension (Ang et al., 2017, 2018; Lockwood et al., 2017). A recent study has also shown that the AMI offers a better specification of emotional apathy than the AES (Petitet et al., 2021).

Regardless, any comprehensive account of apathy must also consider the overlap between social, cognitive, and emotional dimensions that has been observed (Ang et al., 2017; M'Barek et al., 2020) and how these factors might relate to self-awareness. For example, it is possible that executive apathy causes cognitive impairment that also affects our self-awareness, in turn, resulting in indifference to social interactions and emotional neutrality. Asides from self-awareness, other factors are potentially important when considering social apathy, such as those that affect personality. A review of apathy in traumatic brain injury found that self-esteem and beliefs about self-efficacy led to avoidance of challenging situations, of which social interaction is one example (Arnould, Rochat, Azouvi, & Van Der Linden, 2013; Dumont, Gervais, Fougeyrollas, & Bertrand, 2004). This might potentially be important to consider when developing questions to assess social apathy.

## 3 Conclusions

There is overwhelming evidence for the existence of an apathy syndrome. Most researchers are also agreed that apathy is fundamentally a multidimensional syndrome. The last 30 years has witnessed the framing of this syndrome in the context of three main domains that are broadly cognitive, behavioural, and emotional in nature (Levy & Dubois, 2006; Marin et al., 1991; Radakovic & Abrahams, 2018). One difference between these separate apathy frameworks centres on the definition of cognitive apathy: is cognitive apathy a reduction of goal-directed thoughts, or is it more to do with specific problems of executive ability? By endorsing the former argument Marin (1990), a pioneer in the field, has considerably shaped our contemporary understanding of apathy. Indeed, a significant proportion of current apathy scales are representative of Marin's perspective. This despite the general lack of evidence for the three domains he has proposed, even within his own data.

In subsequent analyses, empirical testing of measures of apathy have largely been unable to find evidence for behavioural, cognitive and emotional domains, except perhaps for some studies that have used the LARS (García-Ramos et al., 2014; Sockeel et al., 2006; Yazbek et al., 2014). There have been particular difficulties in convincingly dissociating cognitive and behavioural dimensions. We would suggest that this might in part reflect the fact that “initiative” (cognitive curiosity) and “productivity” (behavioural execution) are not easy to differentiate from the perspective of many apathetic patients or their family members. As such, the distinction between thoughts – being curious – and actions – being motivated to act on that curiosity – is often too complex to tease apart using current questionnaires. On the other hand, an executive account of the cognitive dimension, as proposed by Levy and Dubois (2006), is just as limited. Even if this might help to more easily separate a cognitive dimension from the behavioural one (because executive function can be measured) it is also clear that patients can have deficits in executive function without being apathetic (Marin et al., 2003; Robert et al., 2006) and the opposite also holds true (Brodaty et al., 2010). Apathy can be dissociated from executive dysfunction, and vice versa.

Why then, do so many apathy scales persist with a cognitive or executive account of apathy? We have argued here that in several of the original studies, proper construct analysis is often missing or an unsuitable alternative is used, e.g., PCA, simple correlation analyses, and comparisons to other scales that are similarly unverified or even unidimensional. However, clear behavioural and emotional dimensions are consistently represented across apathy scales, to varying degrees in the LARS (Sockeel et al., 2006), DAS (Radakovic & Abrahams, 2014), and AMI (Ang et al., 2017), unlike crucially for the cognitive dimension. Whether an independent social dimension exists requires further research as this domain has not been addressed in most previous research, although there is now extensive cognitive neuroscience and neurological evidence for distinct brain networks involved in human social interactions (Kennedy & Adolphs, 2012).

## Credit author statement

**Shannon Dickson:** Methodology, Writing – Original draft preparation, Reviewing and Editing. **Masud Husain:** Conceptualisation, Writing – Reviewing and Editing; Supervision.
